# Obesity and anovulatory infertility: A review

**DOI:** 10.5935/1518-0557.20160046

**Published:** 2016

**Authors:** Christiane R Giviziez, Eliane G M Sanchez, Mário S Approbato, Monica C S Maia, Eliamar Aparecida B Fleury, Reinaldo S A Sasaki

**Affiliations:** 1 Universidade Federal de Goiás. Faculdade de Medicina. Hospital das Clínicas. Departamento de Ginecologia e Obstetrícia. Laboratório de Reprodução Humana - Goiânia/GO - Brazil; 2 Unidade Acadêmica de Ciências da Saúde/Biomedicina da Universidade Federal de Goiás, Jataí/GO - Brazil; 3 Faculdade de Ciências Biológicas da Universidade de Rio Verde/GO - Brazil; 4 Seção de Ensino e Pesquisa do Centro de Referência de Medicina Interativa e Complementar/GO - Brazil

**Keywords:** Anovulation, body mass index, female infertility, overweight

## Abstract

This global overweight and obesity epidemics has become one of the largest public health problem worldwide and is increasingly more common among women in reproductive age. Along with the prevalence of overweight women, there is an increase in women with anovulatory infertility. Thus, we carried out a bibliographic research in the PubMed, Lilacs and SciELO databases, using the combinations in Portuguese, Spanish and English of the following descriptors: "Body Mass Index", "obesity", "overweight", "female infertility" and "anovulation". The aim of this study was to assess the effects of obesity on the ovulatory profile of infertile women in the available literature.

## INTRODUCTION

Overweight and obesity have been considered a worldwide epidemics (Loret de Mola, 2009), and in many parts of the world they now represent the greatest threat to public health (Talmor & Dumphy, 2015). In a simplified way, they are defined as the excessive buildup of body fat to such an extent that it becomes detrimental to the health of individuals (WHO, 2015), and its main mechanism is a chronic caloric imbalance between food intake and the body energy expenditure (Sande-Lee & Veloso, 2012). Epidemiological studies are commonly measured by body mass index (BMI), which consists of an estimate of body fat based on height and weight (Haidar & Cosman, 2011). According to the World Health Organization (WHO) (2004), they are considered eutrophics those individuals who have BMI between 18.50 and 24.99 kg/m2, and overweight are those with a BMI between 25.00 and 29.99 kg/m2 and obese are the ones with BMI≥30.00 kg/m2.

There is evidence that, the higher the BMI, the greater the risk of comorbidities such as diabetes mellitus, high blood pressure, dyslipidemia, cardiovascular disease, obstructive sleep apnea, various types of cancer and overall mortality (Hensrud & Klein, 2006). Furthermore, it is believed that a high BMI is also associated with infertility (Castillo-Martinez *et al*., 2003; Parihar, 2003; Bhattacharya *et al*., 2009), especially in what relates to ovulatory disorders in the case of women (Loret de Mola, 2009; Koning *et al*., 2010). It is believed that this group of disorders is responsible for 25 to 50% of causes of female infertility (Weiss & Clapauch, 2014). Ovulatory disorders have clinical manifestations and, occasionally, can cause serious adverse long-term effects (Sadia *et al*., 2009), such as increased risk of endometrial and breast cancer.

One of the main causes of anovulatory infertility is Polycystic Ovary Syndrome (PCOS) (Ben-Shlomo *et al*., 2008). However, not all obese women suffer from PCOS, and not all women with PCOS are obese (ASRM, 2008a; Metwally *et al.*, 2007). Thus, obesity, regardless of PCOS, is associated with anovulation, which shows that other factors contribute to the status of chronic anovulation (Loret de Mola, 2009; Jungheim *et al*., 2012.). Thus, infertility due to ovulatory disorders is a worrying factor, since the prevalence of obesity in women of reproductive age has increased considerably in the last 30 years (Best & Bhattacharya, 2015). All this evidence show that obese women are at an increased risk of developing infertility, and obesity is a major contributor to a variety of etiologies associated with it (Talmor & Dumphy, 2015). However, the way it affects reproduction, including ovulatory disorders, is still an object of investigation (Best & Bhattacharya, 2015). Thus, the aim of this study is to review the literature available in order to assess the effects of obesity in the ovulatory profile of infertile women.

## MATERIAL AND METHODS

We searched papers in PubMed, Lilacs and SciELO databases up to December, 2015, with the combination of the following descriptors in Portuguese, Spanish and English: "Body Mass Index", "obesity", "overweight", "female infertility" and "anovulation". The inclusion criteria were: papers published in English, Spanish or Portuguese until December 2015; papers with key words pre-established in the titles and/or abstracts. Those papers that didn't match the inclusion criteria and those which titles and/or abstracts addressed exclusively the relationship between ovulatory disorders and PCOS were excluded. The papers were preselected after reading their titles and abstracts. After the papers that fit the criteria were spotted, they were read thoroughly. Other references were consulted when necessary, based on references of the analyzed articles.

## RESULTS

### Obesity and anovulation

Puberty, ovulation and reproductive function depend on energy reserves. Weight, body composition, fat distribution and the effects of diet and exercise have been investigated in women with sexual maturation changes, menstrual cycle changes and variations in fertility (Chillik & Grabia, 1999).

In a case-control study, a group of 376 infertile women, who had shown some evidence of ovulatory dysfunction, were compared to fertile women in order to determine if excessive low weight or overweight were associated with an increased risk of infertility. In women who have never been pregnant (nulligravid) (n=204), body weight/height relation ≤ 85% of that "ideal" was associated with an increase of 4.7 fold in the risk of infertility (95% CI 1.5-14.7) associated with ovulatory dysfunction. Nulligravid women who were ≥ 120% above their ideal weight were also at increased risk of ovulatory infertility (RR = 2.1, 95% CI 1.0-4.3) (Green *et al*., 1988). Such results suggest that extremes in body size such as thinness and overweight are a risk factor for ovulatory infertility (Buck *et al*., 1996; Parihar, 2003). It is believed that anovulation occurs more frequently in obese than in normal weight women (Dağ & Dilbaz, 2015; Nelson & Fleming, 2007), and women of reproductive age with high BMI have a higher risk of ovulation problems (Pandey & Bhattacharya, 2010).

Women who have an overweight less than 20% of their body weight have 2.6% of anovulatory cycles. On the other hand, when the percentage of overweight exceeds 74% of their body weight, the number of anovulatory cycles increases to 8.4% (Bray, 1997). Thus, it is believed that the risk of reproductive morbidity, including ovulatory disorders, increases when BMI is also increased (Jungheim *et al*., 2012).

The impact of obesity on reproductive function, especially ovulatory disorders, can be attributed mainly to endocrine mechanisms (ASRM, 2008b), which interfere with neuroendocrine and ovarian functions, and that are able to reduce ovulation rates (Pasquali *et al*., 2003).

A prospective descriptive study was carried out in order to describe the clinical and hormonal profile in subfertile women with ovulatory dysfunction related to BMI. In this study, the clinical criteria were BMI, cervical smear, pelvic ultrasound and hormonal profile. Results suggest that oligomenorrhea, amenorrhea, hirsutism and hormonal changes are more common in infertile patients with overweight than in infertile patients with ovulatory dysfunction with a normal BMI (Sadia *et al*., 2009).

PCOS is the most common endocrine disease in women (ASRM, 2008a) and a major cause of anovulatory infertility (Ben-Shlomo *et al*., 2008). Common clinical characteristics of this disease include obesity, increased WHR and menstrual disorders (ASRM, 2008a). It can be diagnosed, among other factors, by the presence of oligomenorrhea and hyperandrogenism (Jungheim *et al*., 2012). However, not all obese women have PCOS, and not all women with PCOS are obese (ASRM, 2008a; Metwally *et al*., 2007). Thus, obesity, regardless of PCOS, is associated with anovulation, which shows that other factors contribute to the state of chronic anovulation (Bongain *et al*., 1998; Nelson & Fleming, 2007; Loret de Mola, 2009; Jungheim *et al*., 2012).

It is believed that body weight is the major determinant of insulinemia, insulin sensitivity and ovarian hyperandrogenism, independently of PCOS (Dravecka *et al*., 2003). It is, therefore, a consequence of obesity and it is inseparably connected to the same induced anovulation (Malhotra *et al.*, 2013).

Rich-Edwards et al. (2002) observed a U-shaped association between the BMI and the relative risk of ovulatory infertility in North-American women, with an increased risk for BMI lower than 20.0 or higher than 24.0 kg/m2. Based on the distribution of BMI in these women, these results suggest that 12% (95% CI 7-20%) of ovulatory infertility in the United States may be attributable to low weight (BMI<20.0) and 25% (95% CI 20-31%) to overweight (BMI ≥ 25.0).

Researchers from the School of Public Health at Harvard University carried out a long case-control study that found a relationship between BMI at 18 years of age and the subsequent risk of anovulatory infertility. 2,527 women with anovulatory infertility were compared with a control group of 46,718 women, mostly nurses, married, who had recently given birth and did not have a history of infertility. Through a multivariate analysis it was possible to determine the relative risk of anovulatory infertility for different BMI categories: 1.2 (BMI <16), 1.1 (BMI 16 to 17.9), 1.0 (BMI 18 to 19.9), 1.0 (BMI 20 to 21.9), 1.1 (BMI of 22 to 23.9), 1.3 (BMI 24 to 25.9), 1.7 (BMI 26 to 27.9), 2.4 (BMI 28 to 29.9), 2.7 (BMI 30 to 31.9) and 2.7 (BMI ≥ 32). Relative risks for all categories of BMI above 23.9 were significantly elevated. These results suggest that a high BMI at age 18, even at lower levels than those considered overweight, is a risk factor for the subsequent ovulatory infertility (Rich-Edwards *et al*., 1994).

Similar results have been reported in a retrospective study which compared the BMI of 597 women, diagnosed with ovulatory infertility, with 1,695 primiparous controls, who had recently given birth. Obese women (BMI ≥ 27) showed a relative risk of ovulatory infertility at a rate of 3.1 [95% (CI) = 2.2-4.4], compared to those women of lower weight (BMI 20-24.9). A small effect was found in women with a BMI of 25-26.9 or less than 17 [relative risk (RR) = 1.2, CI 95% = 0.8-1.9 and RR = 1.6, CI 95% = 0.7-3.9, respectively). It was concluded that the risk of ovulatory infertility is higher in obese women, but the risk is slightly increased in women with overweight and underweight (Grodstein *et al*., 1994).

Researchers from Saudi Arabia investigated the ovulatory status of 1,755 women. In those with a regular menstrual cycle, ovulatory status was estimated by serial transvaginal ultrasound and progesterone measurements in the second half of the cycle. In those with oligomenorrhea, progesterone measurements were performed one week before the expected onset of menses and those with amenorrhea were considered anovulatory. Among the women that were evaluated, it was found a prevalence of 42% of overweight (BMI 25-29 kg/m2) and 38% of obesity (BMI ≥ 30 kg/m2). With increasing BMI, it was possible to observe that the percentage of oligomenorrhea increased from 18% to 32%, the amenorrhea increased from 2% to 13% and the total percentage of anovulation increased from 32% to 55% (Hamilton *et al*., 1995).

It is believed that the impact of obesity on reproductive function, particularly ovulatory disorders, can mainly be attributed to endocrine mechanisms (ASRM, 2008a); because it interferes with several neuroendocrine and ovarian functions, which are able to reduce the ovulation rate (Pasquali *et al*., 2003).

### Adipose tissue and anovulation

Adipose tissue is the largest endocrine organ of the body and it is involved in several processes, including glucose homeostasis, steroid production, immunoregulation, hematopoiesis and reproduction (Bohler *et al*., 2010); it is also able to convert androgens to estrogens, estradiol to estrone and dehydroepiandrosterone to androstenediol (Parihar, 2003). Thus, the quantity and distribution of adipose tissue can have a significant impact on the reproductive function (Norman & Clark, 1998).

The ability of the adipose tissue to accumulate sex hormones within adipocytes and also to metabolize and interconvert them through local enzymatic reactions can significantly affect the functional status of the reproductive axis. Increased tissue adipocyte mass in obesity may alter the balance between the bioavailability of estrogens, androgens and circulating sex hormones, which altered plasma levels and SHBG could lead to clinical manifestations and dysfunction of the hypothalamic-pituitary-gonadal axis (HPG) (Oliveira & Lemos, 2010).

Most sex hormones seem to concentrate preferentially in the fatty tissue rather than in the blood (Loret de Mola, 2009; Pasquali & Gambineri, 2006). Thus, the steroids pool in obese individuals is higher than what is found in normal-weight individuals (Azziz, 1989).

Another mechanism by which obesity can increase the risk of irregular menses and anovulation is the production of signaling molecules by the adipose tissue, known as adipokines, which appear to have effects on the hypothalamic-pituitary signaling and communication, inhibiting ovulation (Jungheim *et al*., 2012). The increase in body mass and white adipose tissue causes a state of relative hypoxia in groups of adipocytes, causing an inflammatory response, which results in the release of these molecules. Leptin, adiponectin and resistin are among the most studied adipokines that may have an impact on the reproductive performance of obese women (Metwally *et al*., 2008).

Adiponectin, also known as adipocyte complement-related 30 kDa protein (ACRP30), adipoQ, adipose most abundant gene transcript 1 (apM1), and gelatin-binding protein of 28 kDa (GBP28) (Meier & Gressner, 2004) is considered the most abundant gene product in adipose tissue and represents 0.01% of the total plasma protein (Gil-Campos *et al*., 2004). It is especially produced subcutaneously instead of via visceral fat (ESHRE, 2006).

Among the functions of adiponectin, we can include the increased use of fatty acids in lean tissues, inhibition of atherosclerosis, hepatic fibrosis and glucose production by the liver, as well as a potential anti-inflammatory action (Meier & Gressner, 2004; Metwally *et al*., 2008). Those levels are decreased in obesity (Gil-Campos *et al*., 2004; Brewer & Balen, 2010), and that decrease is related to the decrease in insulin sensitivity (Gosman et al., 2006), with RI and hyperinsulinemia (Meier & Gressner, 2004). Thus, adiponectin can provide an important link between obesity, RI and the resultant state of hyperandrogenism (Metwally *et al*., 2007). The low concentration of adipokines in obese women can exert interference on folliculogenesis and modulation of sex steroids secretion (Brewer & Balen, 2010), leading to reproductive disorders.

Leptin, the product of the ob gene, is a recently discovered single-chain proteohormone with a molecular mass of 16 kDa that is believed to play a key role in the regulation of body weight (Friedman & Halaas, 1998). The term leptin is derived from the Greek term leptos meaning "thin". Accordingly, the ultimate aim of leptin is to prevent obesity; which is achieved by inhibition of feeding at the hypothalamic level and increased energy expenditure (Metwally *et al*., 2008).

Thus, leptin acts primarily on the central nervous system, particularly in the hypothalamus, suppressing appetite and stimulating energy expenditure (Webber, 2003). But it seems to play a vital role in the connection between obesity and HPG, regulating the onset of puberty, modulating the reproductive capacity and facilitating the implantation and pregnancy (Oliveira & Lemos, 2010). In relation to the ovary, it is believed that leptin can affect the menstrual cycle through a direct inhibitor effect on the developing follicles. This is grounded in the presence of leptin receptor in the follicular cells. Evidences suggest that leptin influences follicular development and oocyte maturation (Moschos *et al*., 2002; Metwally *et al*., 2008).

Leptin antagonizes the action of insulin and reduces its production in pancreatic β-cells, indirectly affecting the metabolism of glucose (Pasquali & Gambineri, 2006). The hyperleptinaemia associated to obesity may represent an additional factor involved in anovulation, not only by IR induction, but also through a direct impairment of ovarian function (Pasquali, 2006; Pasquali & Gambineri, 2006). Its concentration in the plasma is correlated with body fat content and it is usually increased in obese individuals (Friedman & Halaas, 1998). It means that its concentration increases with increasing adiposity (Lash & Armstrong, 2009). This suggests that human obesity is generally associated with unresponsiveness to leptin (Friedman & Halaas, 1998).

Insulin and leptin may be some of the answers to explain anovulation in obese women (Pantasri & Norman, 2014). It is believed that leptin deficiency can be an independent cause of infertility in these women (Lash & Armstrong, 2009).

Human resistin, a 12.5-kDa protein, contains 108 amino acids of the prepeptide, and its hydrophobic signaling peptide is cleaved before its secretion (Meier & Gressner, 2004). Resistin seems to affect insulin sensitivity in rodents (Bohler *et al*., 2010), and in these, there is a greater amount of it in visceral adipose tissue as opposed to its subcutaneous counterpart (ESHRE, 2006). In humans, its function in IR is not clear yet (Bohler *et al*., 2010). However, it is believed that increased concentrations of resistin may also cause IR (Meier & Gressner, 2004). Thus, hyperinsulinemia in obese women may happen due to the effects of reduced adiponectin levels and increased leptin and resistin in circulation (Gosman *et al.*, 2006; Gil-Campos *et al.*, 2004).

Gosman *et al*. (2006) describe an association between adipokines, adiponectin, resistin and leptin in relation to their effects on insulin sensitivity and possible effects on ovulatory function (Table 1).

**Table 1:** Possible effects of adipokines adiponectin, resistin and leptin on insulin sensitivity and ovulation.

Hormone	Circulating levels in obese	Primary effects	Effect on insulin sensitivity	Possible effect on ovulation
Adiponectin	Decreased	Increases fatoxidation and insulin sensitivity	Increased	Enhanced ovulation
Leptin	Increased	Integrative signal of energy stores, anorexic	Increased	Adverse ovulation
Resistin	Increased	Increases insulin resistance	Decreased	Adverse ovulation

### Body fat distribution and impact on ovulation

There is evidence that there are different hormonal and metabolic responses depending on body fat distribution (Parihar, 2003). The abdominal fat distribution pattern is correlated with increased serum androgens and LH (ESHRE, 2006), which may cause a particular impact on ovulation (Pasquali *et al*., 2007). However, it is not clear whether it is visceral or subcutaneous adipose tissue that is related to reproductive dysfunction (ESHRE, 2006). Visceral abdominal fat buildup has been described as a possible cause of IR and metabolic syndrome (Ogbuji, 2010).

It is believed that women with central body fat have high levels of LH, androstenedione, estrone, insulin, triglycerides, very low-density lipoproteins, and apolipoprotein B, and low levels of high density lipoproteins. These abnormal levels can cause significant disruption in the normal hypothalamic-pituitary-ovarian axis and may result in different gynecologic effects (Parihar, 2003), including ovulatory disorders.

A clinical, descriptive and cross-sectional study was carried out in a group of 41 patients with chronic anovulation and sterility, to correlate IR with body fat composition. Based on BMI, all those patients were overweight or obese, and most of them had a percentage of body fat above the recommended levels. IR was found in 40% of patients. Such disorders were positively associated with the percentage of body fat and androgen distribution. In that study, 73% of the patients had a WHR higher than 0.8; 51.2% were between 0.80 and 0.90, and 22% between 0.91 and 1.28% (Vital Reyes *et al*., 2002).

Móran *et al*. (1999) also reported that upper body obesity appears to affect the ovulation process. They developed a study with 56 women who were overweight or obese (BMI ≥25), in order to determine the effect of body fat distribution in the occurrence of ovulation. The patients were classified into two groups according to the WHR. One with predominance of adiposity in the upper body (n = 29, WHR> 0.85) and another with predominant fat in the lower segment (n = 27, WHR ≤ 0.85). In patients with obesity in the upper body, ovulatory cycles were significantly smaller than those reported for patients with obesity in the lower segment.

A study was carried out in order to compare the distribution of body fat measurements and its contributions to anovulation in infertile obese women, ovulatory and non-ovulatory ones. It was found that obese anovulatory women had higher WC (*P* <0.01), body fat (*P*<0.01) and abdominal fat (*P*<0.05). Data from that study reveals that abdominal fat is increased in anovulatory women due to a significant increase in subcutaneous fat instead of the intra-abdominal one. We came to the conclusion that subcutaneous abdominal fat; and especially the buildup of abdominal and body fat, are associated with anovulation (Kuchenbecker *et al.*, 2010).

Wei *et al.* (2009) carried out a cross-sectional study in which the BMI, WC and WHR of 726 Australian women were obtained, aiming to analyze the association between those measures and the characteristics of their menstrual cycles. Compared with those of normal weight, obese women were at least twice as likely to have an irregular cycle, determined by BMI (odds ratio (OR) = 2.61, CI 95% = 1.28-5.35) , WC (OR 2.28, CI 95% = 1.16-4.49) or WHR (OR = 2.27, CI 95% = 1.09-4.72). In conclusion, both overall and central obesity, were significantly associated with having an irregular menstrual cycle.

## FINAL REMARKS

By performing this study, we discovered that there is a consensus about the negative impact of obesity on anovulatory infertility. Though, how it affects the ovulatory function is still under investigation. Moreover, it was found that most of the studies cited here used BMI as the obesity diagnosis. However, having no standardization of cutoff points for overweight and obesity can hinder comparisons between them. For Maheshwari *et al*. (2010), in addition to those differences found in the definition of a normal BMI, and typical overweight and obesity, the divergences can be explained by the clinical, methodological and statistical heterogeneity of the studies. Another important fact is that in recent years there has been a growing debate about the need to develop different BMI cut-off points for different ethnic groups, due to the growing evidence that the associations between BMI, percentage and body fat distribution differ among populations (WHO, 1995; WHO, 2000). Therefore, it is recommended that further studies be carried out in different ethnic groups to facilitate comparisons. It is believed that the identification of BMI cut-off points that are harmful to ovulatory profile can serve as a tool in the adoption of intervention measures aimed at adapting the necessary weight to improve ovulatory fertility of women who are overweight.

The case of ovulatory dysfunction, specifically anovulation, different methods and parameters used to diagnose ovulation are also factors that can make it difficult to compare results. An example is the determination of serum progesterone (P4), since, in the literature, different cut-off points to determine ovulation are found, ranging from > 3 ng/mL to ≥ 10 ng mL (Rowe *et al*., 1993; Lass, 2005); and it can also cause discrepancies in the results.

In addition to the non-standardization of BMI and P4 cut-off points, it was also found that few studies have been developed with anovulatory women with regular menstrual cycles and who are not PCOS carrier; and this would be important in order to increase our knowledge in this line of research.

Thus, other researches aiming to clarify the impact of obesity on ovulatory profile of infertile women could contribute to the treatment of infertility, since it might facilitate diagnosis by professionals who are involved with the issue and may increase the chance of those women to succeed in trying to get pregnant naturally or by means of assisted reproduction treatment.
